# A cohort evaluation on arterial stiffness and hypertensive disorders in pregnancy

**DOI:** 10.1186/1471-2393-12-160

**Published:** 2012-12-26

**Authors:** Wai Yee Lim, Seang Mei Saw, Kok Hian Tan, George SH Yeo, Kenneth YC Kwek

**Affiliations:** 1 Department of Maternal and Fetal Medicine, KK Women’s & Children’s Hospital, 100 Bukit Timah Road, Singapore, 229899, Singapore; 2 Yong Loo Lin & Saw Swee Hock School of Public Health, National University of Singapore, 10 Kent Ridge Crescent, Singapore, 119260, Singapore; 3 Saw Swee Hock School of Public Health, National University of Singapore, 10 Kent Ridge Crescent, Singapore, 119260, Singapore

**Keywords:** Central aortic pulse pressure, Pulsewave analysis, Pregnancy hypertensive disorders

## Abstract

**Background:**

Hypertensive disorders in pregnancy are associated with systemic endothelial dysfunction leading to impaired physiological vasodilation. Recent evidence has shown central aortic pressures obtained through pulse wave analysis, at less than 14 weeks of gestation, to be predictive of pre-eclampsia. In light of this, we aimed to evaluate the role of central aortic stiffness in the prediction and discrimination of hypertensive disorders in pregnancy.

**Methods:**

A cohort study of women with viable, singleton pregnancies at less than 14 weeks of amenorrhoea, and without multiple pregnancies, autoimmune or renal disease, diagnosed with aneuploidy or fetal anomaly will be recruited from a single maternity hospital and followed up till delivery and puerperium. A targeted sample size of 1000 eligible pregnant women will be enrolled into the study from antenatal clinics. Main exposure under study is central aortic pulse pressure using radial pulse wave recording, and the outcomes under follow-up are gestational hypertension and pre-eclampsia. Other measures include lifestyle factors such as smoking, physical exercise, psychometric evaluations, vasoactive factors, uterine artery pulsatility index, height and weight measurements. These measures will be repeated over 4 antenatal visits at 11-14, 18-22, 28-32 and above 34 weeks of gestation. Double data entry will be performed on Microsoft Access, and analysis of data will include the use of random effect models and receiver operating characteristic curves on Stata 11.2.

**Discussion:**

The proposed study design will enable a longitudinal evaluation of the central aortic pressure changes as a marker for vascular compliance during pregnancy. As measures are repeated over time, the timing and severity of changes are detectable, and findings may yield important information on how aberrant vascular responses occur and its role in the early detection and prediction of hypertensive disorders.

## Background

Hypertensive disorders occur in 10–15% of pregnancies, mainly with gestational hypertension at 10–12%, followed by pre-eclampsia at 3–5% and severe pre-eclampsia at less than 1% [1,2]. About 15% (ranged between 7 to 46%) of women with gestational hypertension, may progress to pre-eclampsia and 9% to severe diseases [3]. Unlike gestational hypertension, preeclampsia is a multi-system disorder involving both maternal and fetal manifestations [4,5].

The origin of pre-eclampsia is rooted in the pathological development of placenta, of which is thought to be associated with angiogenic imbalance [6,7]. Downregulation of placental growth factor (PlGF) and upregulation of soluble fms like Tyrosine Kinase-1 (sFlt-1) have been observed as early as 8 – 10 weeks of amenorrhoea [8] and derangement in these factors are associated with altered vascular endothelial responses in women with pre-eclampsia [9-13]. The relationship between endothelial function, PlGF and sFlt-1 appeared to be consistent [14], and subtle changes in the endothelium have been proposed to occur from first trimester onwards, prior to overt hypertension and proteinuria [6,15].

In the pathological development of gestational hypertension or pre-eclampsia, arterial stiffness or reduced arterial compliance occurs with diffuse vasoconstriction and increased peripheral vascular resistance arising from endothelial dysfunction [6,16]. This led to profound changes in the haemodynamic measures such as the central aortic and peripheral blood pressures. As suggested by Cnossen et al in 2008, brachial blood pressures discriminate poorly in a spectrum of hypertensive disorders. By contrast, central aortic pressures have been associated with better predictive values compared to conventional brachial blood pressures, and as early as less than 14 weeks gestation using pulse wave analysis [17,18]. Moreover, higher arterial stiffness is associated with preeclampsia compared with gestational hypertension or those with normal pregnancies [19].

It has been suggested that combined screening approaches involving ultrasonographic and biochemical markers from first to second trimester performed better than single markers alone in terms of sensitivity and/or specificity [20,21]. In studies with concurrent use of first trimester uterine artery pulsatility index (UAPI), biochemical (PlGF or pregnancy associated plasma protein A) and arterial blood pressure (mean arterial or central augmentation index), there were good detection rate and discriminatory power between variants of hypertensive disorders (gestational hypertension, early and late pre-eclampsia) [22-25]. The simultaneous use of these markers holds promise for early detection of pre-eclampsia as they are indicative of endothelial function and cardiovascular risk.

Although there are evidence to suggest that central aortic pressures are more robust than brachial blood pressures in prediction of cardiovascular outcomes in the general population [26-28], studies in pregnant women are lacking in numbers and sample size (Table 1). Current evidence points to the need for systematic measurements of central aortic pressures across pregnancy trimesters till the development of hypertension, and the adjustment for potential confounders. Study findings may unravel how the development of aortic stiffness in hypertensive disorders in pregnancy occurs, and their potential value if any, beyond brachial blood pressures in clinical application.

**Table 1:** Studies evaluating central aortic pressures on pregnancy associated hypertensive disorders

**ID**	**Author year/country published**	**Study design/sample size**	**Study population (Stage of pregnancy)**	**Measures of arterial stiffness**	**Normal pregnancy group**	**Pre-eclampsia group**	**P Value (Crude analysis)**
1	Khalil [10] 2008 United Kingdom	Cohort 218	11–13 + 6 weeks of amenorrhea	AI	AI-OR1(Referent)	AI-§ Odds Ratio 21.3 (95% CI5.5-82.3)	<0.001
2	Robb [15] 2010 United Kingdom	Cohort 47	At 16 weeks of amenorrhea/after PE diagnosis	CAPP	CAPP-Mean 26 (SD1) mmHg	CAPP- Mean 37 (SD2) mmHg	<0.0001
3	Spasojevic [8] 2005 Australia	Cohort 99	24-39 weeks of amenorrhea	AI	AI-Mean 0.3(SD7.4)%	AI-Mean 24.2(SD8.1)%	<0.0001
4	Khalil [9] 2009 United Kingdom	Nested case control 252	11 – 13 + 6 weeks of amenorrhea	AI	AI-ǂ MOM 0.8 (IQR0.5-1.2)%	AI-ǂ MOM 1.5 (IQR1.3-1.8)%	<0.002
5	Avni [14] 2010 United States of America	Cross-sectional 100	Any pregnancy gestation	AI	AI-Mean 12.3 (SD7.4)%	AI-Mean 32.4 (SD7.5)%	0.001
6	Kaihura [16] 2009 United Kingdom	Cross-sectional 123	Any pregnancy gestation	CAPP & AI	CAPP-Median 27 (IQR24.2-31.5) mmHg	CAPP-Median 43.5 (IQR37.7-47.8) mmHg	<0.0001
					AI-Mean 4 (SD13.6)%	AI-Mean 25.1 (SD11.2)%	<0.0001
7	Ronnback [17] 2005 Finland	Cross-sectional 96	30-42 weeks of amenorrhea	CAPP & AI	CPP-Mean 29 (Range 23-45) mmHg	CPP- Mean 39 (Range 28-65) mmHg	<0.001
					AI-Mean 4 (SD1)%	AI-Mean 23 (SD2)%	<0.001
8	Taspinar [18] 2004 Netherlands	Cross-sectional 122	Third Trimester	CAPP & AI	CPP-Mean 28 (SD5) mmHg	CPP- Mean 44 (SD17) mmHg	<0.05
					AI-Mean 6.7 (SD14)%	AI-Mean 31.1 (SD12.4)%	<0.05

CAPP = Central Aortic Pulse Pressure in mmHg, AI = Augmentation Index in%,

* = Median (Interquartile range).

ǂ = Multiples of Median(Interquartile range).

§ = Odds Ratio(95% confidence interval).

### Study aims

This study aims to evaluate the role of central aortic stiffness in prediction of hypertensive disorders in pregnancy. The specific objectives are:

1. To identify gestation specific factors associated with central aortic stiffness as measured by central aortic pulse pressure (CAPP).

2. To measure the association between central aortic stiffness as quantified by CAPP and pregnancy associated hypertensive disorders

3. To determine the predictive performance of CAPP, sFlt-1 and PlGF, and UAPI measured at first trimester for early detection hypertensive disorder in pregnancy

## Methods

### Study design and setting

A prospective cohort of 1000 pregnant women will be recruited for follow-up till delivery and puerperium in KK Women’s and Children’s Hospital, a maternity hospital which handles an average of 12000 deliveries annually. This study has been approved by Institutional Review Board in 2010. Written informed consent will be obtained from all study participants.

### Study population and sampling method

The study population will comprise mostly of Chinese, Malay and Indian ethnicities following the make-up of Singapore resident population [29]. Women with viable, singleton pregnancies at less than 14 weeks of amenorrhoea will be recruited, and those with multiple pregnancies, autoimmune or renal disease, diagnosed with aneuploidy or fetal anomaly will be excluded.

**Figure 1:**
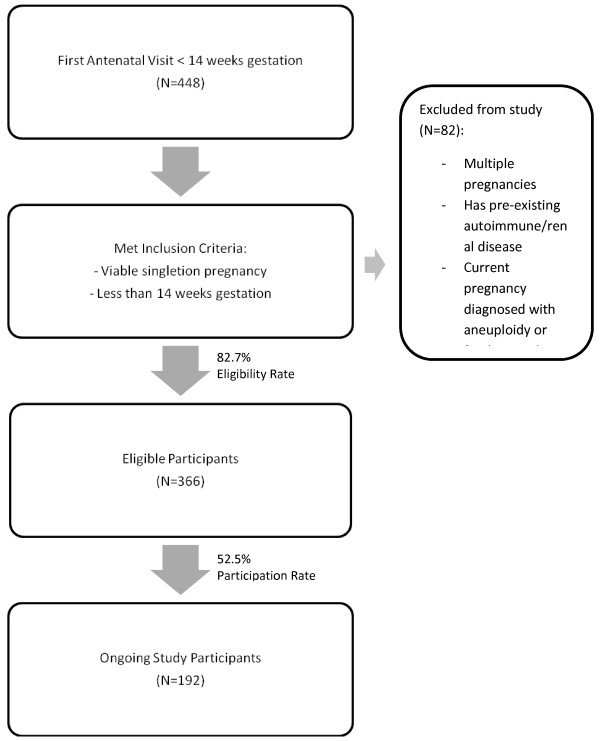
Target sample at antenatal outpatient clinics (study status as of November 2011).

A convenient sampling of women seeking antenatal care at the outpatient clinics will be screened for study recruitment. Based on 80% eligibility and 50% participation rates, the target sample size of 1000 out of 2400 patients is attainable (Figure 1). The final sample size is expected at 850 having accounted for 15% dropout rate.

### Sample power calculation

Assuming a 10% difference in incidence of hypertensive disorder between the lowest and highest quartiles of central aortic pulse pressure, and a base incidence of 5%, a total of 207 participants are required in each quartile to achieve 80% power and at 5% alpha. The overall study sample therefore is at 828 participants with complete study follow-ups.

### Recruitment strategies

Recruitment began in April 2011, at the antenatal specialist outpatient units and staffs at these units were briefed on the project. At screening, basic demographic factors such as education, employment and marital status are collected. Women who are unsure about study participation will be contacted within the week, and after 3 unsuccessful attempts of telephone contact within 2 weeks; patient will be deemed ‘uncontactable’. Women who had consented into the study, and subsequently miscarried or decided to deliver elsewhere will be considered as ‘ineligible’.

### Study procedures

After written consent, the study participants will be followed-up till the diagnosis of hypertensive disorder in pregnancy (gestational hypertension or pre-eclampsia) or delivery. Central aortic blood pressures being the exposure of interest will be measured over 4 study visits at 11-14, 18-22, 28-32 and above 34 weeks of gestation. Study follow-up for outcome ascertainment will continue till hospital discharge after delivery.

**Figure 2:**
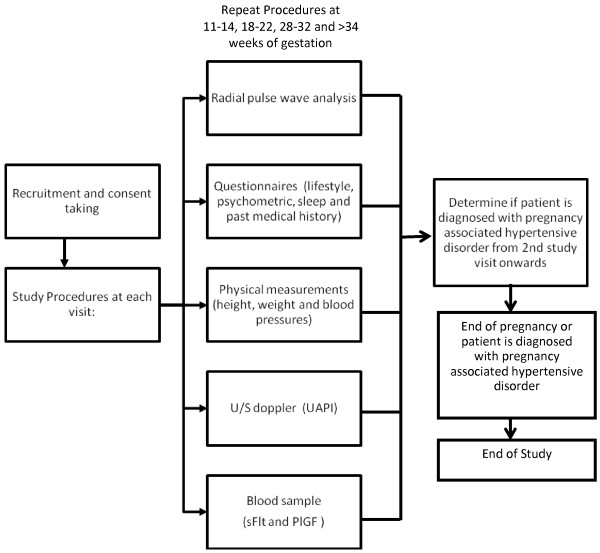
Flowchart for study procedures.

At study enrolment, baseline information on socio-economic, personal medical and obstetric history will be obtained through an interviewer administered questionnaire. Other measures involving lifestyle behaviours, psychometric assessments and biophysical measures such as anthropometry, UAPI and blood sample for vasoactive factors (sFlt-1 and PlGF) will be done simultaneously at the 4 study visits as mentioned above (Figure 2).

### Central aortic pulse pressure measurement

Central aortic pressures are derived from radial pulse wave analysis, obtained through a BPro® device. It is a wrist watch like instrument which utilizes applanation tonometry on the radial pulse, developed by HealthStats, Singapore. The study participants are required to abstain from coffee intake for at least 0.5 hour, and prior to measurement, they are to be seated comfortably and rested for at least 5 minutes in a quiet room. From the right upper arm, brachial blood pressures are repeated three times at intervals of 30 – 60 seconds using an oscillometric device by HealthStats (MC3100), whereby difference between each measurement should not differ by more than 10 mmHg for either systolic or diastolic pressures. After achieving a stable reading, the systolic and diastolic averages will be used to calibrate the radial pulse wave forms and continuous sampling of the latter will ensue for 40-60 seconds.

Central aortic pulse pressure as a measure of arterial stiffness is the difference in pressure between central aortic systolic and brachial diastolic pressures in mmHg. This measure is dependent on the use of radial pulse wave analysis to derive central aortic systolic pressure which has shown good agreement with invasive measurements using cardiac catheterization [30], and that diastolic pressure is stable between central and peripheral (brachial) sites [31,32]. An internal validation has been conducted for the measurement of central aortic pulse pressure, and the coefficient of variation is at 1.9%.

### Other measures

Questionnaires are employed to gather information pertaining to socio-economic, lifestyle, sleep and maternal coping during pregnancy. Interviewer administered structured questionnaires were used to collect demographic data on variables such as maternal and partner’s date of birth, race, education, occupation, type of housing, household size and income; life style variables such as alcohol and coffee intake, physical exercise and daily activities and smoking habits; and lastly personal or family history of gestational diabetes, hypertension or pre-eclampsia.

Validated self-administered questionnaires are used to determine sleep and maternal coping in pregnancy. Assessment of sleeping habit are done through Pittsburgh Sleep Quality Index [33]; and maternal coping during pregnancy through psychometric assessments on maternal stress, anxiety and depression scores using Stress Trait & Anxiety Inventory [34], Perceived Stress Scale [35], Roesch Questionnaire [36] and Edinburgh Postnatal Depression Scale [37] respectively.

All self-administered questionnaires are available in Chinese and Malay versions. These questionnaires are translated forward and backward into English for all except Stress Trait & Anxiety Inventory, as it is available in both languages. Both interviewer and self-administered questionnaires are administered by trained staffs that are conversant in both English and Chinese or Malay.

Biophysical measurements including anthropometry, ultrasound doppler (UAPI) and serum biomarkers for sFlt-1 and PlGF are done by hospital nurses, sonographers and laboratory technologists that are accredited to perform these procedures. Serum samples would be aliquoted and stored in -80°C at the research laboratory.

### Assessment of hypertensive disorder in pregnancy

The definitions of hypertensive disorder in pregnancy are based on the criteria as specified by Working Group on High Blood Pressure in Pregnancy [4,38]. Therefore, gestational hypertension is defined as new onset of hypertension in pregnancy with brachial systolic pressure at 140 mmHg and above, or a diastolic pressure of 90 mmHg or above on repeated measurements of more than 6 hours interval within 1 week after 20 weeks gestation without proteinuria. In pre-eclampsia however, the definition includes both hypertension and proteinuria at above 0.3 g protein/24 h (or in emergency cases only a dipstick >1+ on more than one occasion or ≥ 30 mg/dL protein in a spot urine if a 24 hour urine protein collection cannot be obtained). Outcome ascertainment will be done by trained medical personnel based on the above criteria.

### Data management

Data entry will be done by 2 independent research staff in 2 separate access databases. Weekly entry of raw data will be done to ensure timely clearance. At data verification, databases will be locked and no entries are made until all queries are resolved. Training and simulations for data entry have been conducted for familiarization purposes. Moreover, protocols for data entry, verification and cleaning are provided to staff involved in data entry and cleaning.

### Statistical methods

At the end of data validation and cleaning, data will be prepared for analysis through reduction of some variables into fewer categories such as occupation and generation of new variables such as body mass index. Data analysis will be carried out using Stata version 11.2 (Statacorp, College Station, Texas). Analysis will include distribution of sample characteristics in relation to gestational hypertension and pre-eclampsia in proportions and central tendencies; and their crude associations using mixed models for correlated data.

Selection of potential risk factors will be based on biological plausibility and at a significance level of 0.2 for subsequent modelling. Receiver Operator Characteristic curves will be employed to assess the predictive capabilities of tests. Sensitivity and likelihood ratios will be reported based on 5% and 10% false positive rates. Missing data will be assessed for bias and depending on its nature, sensitivity analysis or multiple imputation techniques may be employed to deal with the loss of information in the dataset.

## Discussion

Recruitment of study participants begun in April 2011 and by November 2011, 366 eligible pregnant women were screened and 192 (52.5%) had consented into the study. The recruitment rate was slower than expected as recruitment was temporally suspended from late August 2011 to end October due to staffing issues. During this period, study follow-ups were on-going to minimize disruption to study flow. Recruitment resumed in early November, with 2 research coordinators actively screening for eligible participants at antenatal outpatient clinics.

Amongst the 192 study participants, 84 (44%) were Chinese, 60 (31%) Malays, 19 (10%) Indians and 29 (15.1%) were of other ethnicities. The mean age was at 30 years old, ranging from 19 to 44 years old. About 47 (25%) received tertiary education, and 138 (73%) were employed at the time of recruitment. Preliminary comparison between those who consented and refused study participation showed that age, education level and employment status were similar. However, there were significant racial differences amongst those who refused participation, whereby Indians tended to refuse participation (64.2%) and Malays tended to participate (60.6%), p value = 0.036.

From literature review, elevations in central aortic pressures (augmentation index, systolic and diastolic pressures) are greatest amongst women with pre-eclampsia, followed by gestational hypertension compared to women with normal pregnancies [16,39-43]. Predictive performance using central aortic augmentation index measured at 11-14 weeks of amenorrhoea has 50-57% sensitivity in detecting pre-eclampsia [17,18] as compared to 24-35% for brachial systolic, diastolic and mean arterial blood pressures [15].

Although current reports on these measures provide valuable information about circulatory changes associated with hypertension disorders in pregnancy, presently they do not infer central aortic pressures ability to discriminate pre-eclampsia from gestational hypertension or normal pregnancies. The observed difference in central aortic pressures may be flawed due to the cross-sectional approach that utilized concurrent measurement of central aortic pressures and pre-eclampsia [44]. It is therefore difficult to establish temporality i.e. elevation of central aortic pressures prior to pre-eclampsia.

Furthermore, there are limitations associated with interpretation of these indices. Firstly, the validity of the central aortic augmentation index is questionable as they were derived from a generalized transfer function which assumed similar baseline characteristics for its subjects [45-47]. To date, only central aortic systolic pressure has been shown to have excellent correlation and agreement with direct measurement of aortic root pressure during cardiac catheterization [30]. Secondly, inadequate adjustment for factors that affect central aortic pressures may possibly invalidate the reported difference as observed between pre-eclampsia, gestational hypertension and normal pregnancies [39-41,43]. Lastly, as most measures were taken at one singular event at 2^nd^ trimester onwards, the onset and magnitude of insult i.e. endothelial dysfunction leading to arterial stiffness is uncertain.

Therefore, the rationale of the study aim is to systematically measure changes in central aortic stiffness and associated factors beginning from first trimester onwards to account for gestation specific physiological changes as well as for alterations in environmental exposures and shifts in the mental and emotional states of pregnant women, prior to the development of gestational hypertension or pre-eclampsia. Secondly, central aortic pulse pressure is used as a measure of central aortic stiffness instead of central augmentation index, as it is a direct derivation from the difference between central aortic systolic and diastolic pressures, without the assumptions of a generalized transfer function as applied to the central aortic augmentation index.

The cohort design will resolve issues associated with temporality as reported in most studies using cross sectional evaluation. However, losses to follow-up may potentially introduce attrition bias into the study. Therefore retention of study participants will be important and this may be achieved through rapport building and close communication with the study participants. Selection bias may occur due to the convenient sampling technique and an analysis on the socio-economic variables of women who refused study participation to evaluate the occurrence of selection bias will be done.

In conclusion, as raised arterial pressure is a manifestation endothelial dysfunction, targeted measurement of central aortic pressures in this study may yield important information how these aberrant responses occur and its role early detection and prediction of hypertensive disorders in pregnancy.

## Competing interests

The authors have no competing interests.

## Authors’ contributions

KK, KH, GY and WY led the conceptualization and design of the original project, prepared the proposal and obtained funding. WY, SM and KK developed this current protocol and manuscript with input from KH and GY. All authors reviewed and approved the final version of manuscript.

## Authors’ information

WY: BNurs, Dip LSHTM, MSc Epidemiology. This protocol was written as part of the PhD requirement.

SM: MBBS, MPH, PhD, FAMS. Professor of Epidemiology and Ophthalmology. Vice Dean (Research), Yong Loo Lin School of Medicine

KK: MBBS, M Med (O&G), MRANZCOG, MRCOG Associate Professor, Senior Consultant, Head of Maternal and Fetal Department and Chief Executive Officer of KK Women’s & Children’s Hospital

KH: MBBS, MRCOG, M Med (O&G), FAMS, FRCOG Associate Professor, Senior Consultant, Division Chairman (O&G), Head of Perinatal Audit and Epidemiology

GY: MBBS, FRCOG, FAMS Professor, Chief of Obstetrics, Head & Senior Consultant of Obstetric Ultrasound and Prenatal Diagnosis Unit

## Pre-publication history

The pre-publication history for this paper can be accessed here:

http://www.biomedcentral.com/1471-2393/12/160/prepub
